# Preparation of PEG/ZIF-8@HF drug delivery system for melanoma treatment via oral administration

**DOI:** 10.1080/10717544.2022.2058649

**Published:** 2022-04-04

**Authors:** Luxi Peng, Jiajun Qiu, Lidan Liu, Xiaoyu Li, Xuanyong Liu, Yongjun Zhang

**Affiliations:** a The Third Affiliated Hospital of School of Medicine, Shihezi University, Shihezi, China; b The State Key Lab of High Performance Ceramics and Superfine Microstructure, Shanghai Institute of Ceramics, Chinese Academy of Sciences, Shanghai, China; c Department of Pharmacy, Zhongshan Hospital, Fudan University, Shanghai, China

**Keywords:** Hinokiflavone, melanoma, zeolitic imidazolate framework-8, cytocompatibility, antitumor

## Abstract

Melanoma is one of the highly malignant tumors whose incidence and fatality rates have been increased year by year. However, in addition to early surgical resection, there still lacks specific targeted drugs and treatment strategies. In this study, it was discovered that hinokiflavone (HF) encapsulated in zeolitic imidazolate framework-8 (ZIF-8) exhibited a superior anti-melanoma effect *in vitro* and *in vivo*. HF was encapsulated in ZIF-8 through a one-step synthesis method, and polyethylene glycol (PEG-2000) was used to optimize the size and dispersion of the drug-loaded complex (PEG/ZIF-8@HF). The results show that the prepared PEG/ZIF-8@HF has a high encapsulation efficiency (92.12%) and can achieve selective drug release in an acidic microenvironment. The results of *in vitro* anti-melanoma experiments indicate that PEG/ZIF-8@HF shows up-regulation of reactive oxygen species (ROS) levels and can restrain the migration and invasion of B16F10 cells. Moreover, *in vivo* animal experiments further confirm that PEG/ZIF-8@HF shows anti-tumor effect by up-regulating the pro-apoptotic proteins caspase-3 and caspase-8, and down-regulating the migration-promoting invasion protein MMP-9. This study developed a safe and effective oral administration of HF based on the high-efficiency delivery ZIF-8 system, which provides an effective treatment strategy for melanoma.

## 1. Introduction

Melanoma is one of the most common forms of skin cancer causing 80% of skin cancer-related deaths (Gray-Schopfer et al., 2007; Castro et al., 2021). In recent years, melanoma incidence and fatality rates at home and abroad have increased which ranks fifth among common cancers in the USA (Miller et al., 2020). It is the third most common tumor in Australia (Scolyer et al., 2013). Once melanoma spreads, it will quickly endanger patients' lives and the available treatment options are tiny. Approximately, 55,500 people die of melanoma every year worldwide, accounting for 0.7% of cancer mortality (Schadendorf et al., 2018).

In recent years, surgical resection, immunotherapy, targeted therapy, chemotherapy, radiotherapy, soft X-rays, and photodynamic therapy are widely used for melanoma treatment (Davids & Kleemann, 2011; Koller et al., 2017; van Zeijl et al., 2017; Yaman et al., 2020). Nevertheless, the therapeutic effect of surgery on melanoma is limited, and traditional drugs such as doxorubicin and etoposide are highly resistant and often cause a variety of side effects (Soengas & Lowe, 2003; Pugazhendhi et al., 2018). Immunotherapy has a particular curative effect on melanoma, but its high cost and complexity significantly limit its clinical application (Del Paggio, 2018; Zhou et al., 2020; Li et al., 2021; Zhang et al., 2022). Hence, it is necessary to develop a novel anticancer agent with low side effects and significant curative effects for melanoma.

Among the bisflavonoids extracted from Selaginella, hinokiflavone (HF) has an ether bond structure between the two apigenin units of apigenin and luteolin subunit, which is more stable and has the potent anti-tumor activity (Wang et al., 2018). Current studies have shown that HF can inhibit the RNA splicing, suppress the proliferation, migration, and invasion of cancer cells, activate the mtROS/JNK/caspase pathway and inhibit the NF-κB signaling pathway to induce cell apoptosis (Pawellek et al., 2017; Zhou et al., 2019; Mu et al., 2020). However, HF is difficult to dissolve in water, resulting in low bioavailability and high oral dose, which greatly limits its clinical application (Chen et al., 2019).

Chen et al. synthesized a HF-loading system using soluplus, d-α-tocopherol acid polyethylene glycol 1000 succinate, and dequalinium by a thin-film hydration method to load HF into hybrid micelles for anti-lung adenocarcinoma, but its drug loading rate was not satisfactory (Chen et al., 2020). Metal-organic frameworks (MOFs) are widely used in drug delivery fields due to their high specific surface area, nano-scale size, and easy functional modification (Abánades Lázaro & Forgan, 2019; Yang & Yang, 2020). Among them, zeolitic imidazolate framework-8 (ZIF-8) has good stability, adjustable pore size, biocompatibility, and slightly acidic responsive disintegration characteristics, which can be used as an excellent drug delivery material (Li et al., 2016, 2018, 2020).

In this work, HF was encapsulated into ZIF-8 and grafted with polyethylene glycol (PEG/ZIF-8@HF). PEG/ZIF-8@HF shows good water solubility, high drug loading (20.94%), and encapsulation efficiency (92.12%). PEG/ZIF-8@HF can realize drug release in response to an acidic microenvironment. Both *in vitro* and *in vivo* experiments have indicated that PEG/ZIF-8@HF shows anti-tumor activity against melanoma.

## 2. Experimental

### 2.1. Materials

2-Methylimidazole (Hmim) and Zn(NO_3_)_2_·6H_2_O were purchased from Aladdin Reagent Inc. (Shanghai, China). PEG-2000 was purchased from HUSHI (Shanghai, China). Hinokiflavone (purity >97%) was purchased from Chengdu Herbpurify Co., Ltd. (Chengdu, China). Methanol (purity ≥ 99.5%) was purchased from Shanghai Lingfeng Chemical Reagents Co., Ltd. (Shanghai, China). The reagents are utilized without further purification. The ultrapure water is prepared using the Millipore purification system (Billerica, MA).

### 2.2. Preparation of PEG/ZIF-8@HF

The drug loading scheme was optimized by comparing the drug loading rate and encapsulation rate under different process conditions. PEG/ZIF-8@HF was prepared by one-step synthesis method. In details, 330 mg of Hmim, 480 mg of PEG-2000, and 10 mg of HF were co-dissolved in 9.9 mL methanol at 20 °C, and then 100 µL of dimethylsulfoxide (DMSO) was added. At the same time, 150 mg of Zn(NO_3_)_2_·6H_2_O was dissolved in 5 mL of ultrapure water and then transferred to the Hmim solution under stirring. Finally, PEG/ZIF-8@HF was obtained after centrifuging the solution at 12,000 rpm for 0.25 h using a centrifuge (Thermo Fisher Scientific X1R, Waltham, MA). ZIF-8, ZIF-8@HF, and PEG/ZIF-8 were prepared using the same method as aforementioned for comparison. The absorbance at 337 nm of supernatant was detected with a Lambda750 Ultraviolet Spectrophotometer (PerkinElmer, Waltham, MA). According to the standard curve of absorbance and mass, the content of HF was obtained. At last, the drug-loading and encapsulation efficiency of HF were calculated according to the reference Chen et al. (2020).

### 2.3. Sample characterization

Surface morphologies of PEG/ZIF-8 and PEG/ZIF-8@HF were observed using a transmission electron microscope (TEM, FEI Electron Optics G2 F20, Eindhoven, Netherlands). Malvern laser particle size analyzer (Zetasizer Nano ZS, Malvern, UK) was utilized to analyze the particle sizes, polydispersity index (PDI), and zeta potentials. X-ray diffraction (XRD, D2 Phaser, Bruker, Ettlingen, Germany) with Cu Kα radiation (*λ* = 0.154 nm) was used to detect the phase structure of PEG/ZIF-8 and PEG/ZIF-8@HF.

### 2.4. Release behaviors of HF from PEG/ZIF-8@HF under different pH conditions

PEG/ZIF-8@HF was dispersed in phosphate buffer solution (PBS) containing 10% fetal bovine serum (FBS, Gibco, Mulgrave, Australia) with different pH values (pH = 7.4, 6.8, and 5.5) under shaking with 100 rpm at 37 °C for 2 h, 4 h, 8 h, 16 h, and 24 h. At each time point, 1 mL of the release solution was collected and centrifuged to take the supernatant, and the released amount of HF was measured. The release rate is calculated as follows: release rate (%)=Mr/Mt, where Mr indicates the release amount of HF and Mt represents the total load of HF.

### 2.5. *In vitro* biocompatibility evaluation

#### 2.5.1. Cell culture

Mouse fibroblast cells (L929) and mouse melanoma cells (B16F10) were acquired from the Cell Bank of Chinese Academy of Sciences (Shanghai, China). L929 cells and B16F10 cells were cultured with α-minimum essential medium (α-MEM, Gibco, Invitrogen Inc., Carlsbad, CA) and Roswell Park Memorial Institute (RPMI) 1640 Medium (1640) (Gibco, Carlsbad, CA), respectively, containing 10% FBS (Gibco, Carlsbad, CA) and 1% penicillin–streptomycin (Gibco, Carlsbad, CA) in an incubator at 37 °C and 5% CO_2_.

#### 2.5.2. Cell proliferation

The alamarBlue (Gibco, Carlsbad, CA) assay was utilized to assess the cell proliferation. In short, L929 and B16F10 cells were introduced into a 96-well plate at a cell concentration of 1.0 × 10^4^ cells/well and cultured for 24 h. Then, PEG/ZIF-8, HF, and PEG/ZIF-8@HF were introduced and cultured for another 24 h. Finally, the cell culture medium was discarded and 100 μL of fresh culture media containing 10% (v/v) alamarBlue were introduced and incubated at 37 °C for 2 h. Then, fluorescence intensity of the aforementioned solution was measured at an excitation wavelength of 560 nm and an emission wavelength of 590 nm using Cytation 5 Multi-Mode Reader (BioTek, Winooski, VT).

#### 2.5.3. Live/dead cell staining

The live/dead cell staining kit (Thermo Fisher Scientific Inc., Waltham, MA) was utilized to assess the cell viability of L929 cells incubated with PEG/ZIF-8, HF, and PEG/ZIF-8@HF. First, L929 cells were introduced into a 96-well plate with the cell concentration of 1.0 × 10^4^ cells/well and cultured for 24 h. Then, PEG/ZIF-8, HF, and PEG/ZIF-8@HF with HF content of 7.5 μM were cultured with the cells for 24 h, and this concentration was also used in subsequent experiments. Then, the culture media were discarded and the cells were rinsed with PBS. Subsequently, 60 μL of culture medium containing propidium iodide (5 μM) and calcium-AM (2 μM) was introduced into each well and incubated for 0.5 h. At last, the cells were observed with a fluorescence microscope (Olympus, Tokyo, Japan).

#### 2.5.4. Mitochondrial reactive oxygen species (ROS) levels analysis

The 2′,7′-dichlorodihydrofluorescein diacetate (DCFH-DA, Sigma, St. Louis, MO) fluorescent probe was used to detect the ROS levels produced by PEG/ZIF-8, HF, and PEG/ZIF-8@HF. In details, control (without sample), PEG/ZIF-8, HF, and PEG/ZIF-8@HF were cultured with B16F10 cells for 6 h. Subsequently, the cells were rinsed with PBS, and DCFH-DA solution was introduced and cultured for 0.5 h. At last, the fluorescence images were taken with a fluorescence microscope (Olympus, Tokyo, Japan).

#### 2.5.5. Cell migration assay

When the cells grow to more than 90% fusion, a 100 μL pipette tip was utilized to streak evenly on L929 and B16F10 cells in a 24-well plate. Then, samples were introduced and cultured for 12 h. Then, the cells were fixed with 4% paraformaldehyde (PFA, Gibco, Carlsbad, CA) solution and stained with DAPI (Akoya Biosciences, Marlborough, MA). Fluorescence images were taken by a fluorescence microscope (Olympus, Tokyo, Japan).

#### 2.5.6. Transwell tumor cell migration and invasion experiment

Transwell tumor cell migration was performed according to the Boyden chamber migration assay in the literature with some modifications (Kramer et al., 2013). In short, first, B16F10 cells were cultured with various samples using the medium with 5% FBS for 24 h. Subsequently, B16F10 cells were collected and diluted to 1.0 × 10^5^ cells/mL with the medium containing 5% FBS. Subsequently, 300 μL of the diluted cell suspensions was introduced into the upper chamber, 600 μL of culture medium containing 20% FBS was introduced into the bottom and cultured for 24 h. At last, the cells in the upper chamber were washed with PBS, fixed with 4% PFA, and stained with 1% crystal violet. Finally, photographs were taken and the number of cells was counted. The cell invasion assay was carried out according to the literature with some changes (Ye et al., 2014). Specifically, 40 μL solution of serum-free culture medium and Matrigel (Discovery Labware, Inc., Bedford, MA) with the volume ratio of 8:1 was introduced into the upper chamber, and cultured for 0.5 h to form a gel and then aspirate the ungelled part. Subsequently, 600 μL of culture medium containing 20% FBS was introduced into the lower chamber. Then, 300 μL of cell suspensions pretreated as mentioned in cell migration experiment was introduced into the upper room and cultured for 24 h. Finally, the cells in the upper chamber were fixed with 4% PFA, and stained with 1% crystal violet. Photographs were taken and the number of cells were counted.

#### 2.5.7. In vivo anti-melanoma effect evaluation

The animal experiments have been approved by the Ethics Committee of the First Affiliated Hospital of School of Medicine, Shihezi University and comply with all Chinese laws and regulations on animal welfare. The BALB/c female nude mice were acquired from Zhejiang Vital River Laboratory Animal Technology Co., Ltd. (Beijing, China) and raised in an SPF environment. The temperature was kept at 22 ± 1 °C, the humidity was maintained at 65–70%, and the light/dark light cycle was 12 h. Thirty-two nude mice were randomly divided into four groups (PBS, PEG/ZIF8, HF, and PEG/ZIF-8@HF). After one week of adaptive feeding, the B16F10 cells were diluted with PBS to 4.0 × 10^6^ cells/mL, and 100 μL of the cell suspension was injected subcutaneously into the abdominal wall of each nude mouse to establish a B16F10 xenograft tumor model. When the tumor volume was grew to ∼100 mm^3^, the same dose of PBS, PEG/ZIF8, HF, and PEG/ZIF-8@HF (80 mg/kg HF) was administered intragastrically on 0, 2, 4, 6, 8, and 10 days, and corresponding tumor volume and weight of nude mouse were recorded. On the 14th day, four groups of nude mice were sacrificed, and the tumors were excised to evaluate the tumor inhibitory effect. The main organs including heart, liver, spleen, lung, and kidney were collected. Part of tumor tissues was used for Western blot detection after protein extraction. Part of tumors was fixed in 10% formalin solution, followed by immunohistochemistry, H&E staining, and TUNEL detection.

### 2.6. Statistical analysis

All the data are presented by the mean ± standard deviation (SD). The two-way analysis of variance is utilized for the evaluation of statistical analysis. The *p* value <.05 suggests statistically significant.

## 3. Results and discussion

### 3.1. PEG/ZIF-8@HF preparation

In this study, a single factor test method was utilized to assess the effects of solvent, synthesis temperature, and PEG/Hmim ratio on the drug loading and encapsulation efficiency of PEG/ZIF-8@HF NPs. Table 1 shows that drug loading of 7.79% and encapsulation efficiency of 43.29% are obtained when PEG/ZIF-8@HF was synthesized with water as a solvent, while drug loading of 20.94% and encapsulation efficiency of 92.12% are obtained when PEG/ZIF-8@HF was synthesized in mixed solvent of water and methanol with the volume ratio of 2:1. It suggests that a higher drug loading and encapsulation efficiency can be gained in a mixed solution with water and methanol. As shown in Table 2, drug loading of PEG/ZIF-8@HF synthesized at 10 °C, 20 °C, 30 °C, and 60 °C is 9.01%, 20.94%, 16.43%, and 19.47%, respectively. While encapsulation efficiency of PEG/ZIF-8@HF synthesized at 10 °C, 20 °C, 30 °C, and 60 °C is 60.75%, 92.12%, 87.05%, and 59.37%, respectively. It can be seen that PEG/ZIF-8@HF synthesized at 20 °C shows a higher drug loading and encapsulation efficiency. Therefore, 20 °C is selected as the synthesis temperature. It can be seen from Table 3 that drug loading of 12.46%, 20.94%, and 15.89% is obtained which corresponds to the PEG/Hmim ratio of 3%, 6%, and 12%, respectively. Additionally, encapsulation efficiency of 82.87%, 92.12%, and 85.78% corresponding to the PEG/Hmim ratio of 3%, 6%, and 12%, respectively, are gained. It suggests that drug loading and encapsulation efficiency of PEG/ZIF-8@HF present the highest value at the PEG/Hmim ratio of 6%. Therefore, mixed solvent with water and methanol (volume ratio is 1:2), PEG/Hmim ratio of 6%, temperature of 20 °C were used to synthesize PEG/ZIF-8@HF for the follow-up experiments.

**Table 1.** The effect of synthetic solvents on drug loading.

Synthetic solvent	Drug loading (%)	Encapsulation efficiency (%)
Water	7.79 ± 0.52	43.29 ± 2.85
Water:methanol = 1:2	20.94 ± 0.01	92.12 ± 0.03

**Table 2.** The effect of synthetic temperature on drug loading.

Synthesis temperature (°C)	Drug loading (%)	Encapsulation efficiency (%)
10	9.01 ± 0.04	60.75 ± 0.21
20	20.94 ± 0.01	92.12 ± 0.03
30	16.43 ± 0.02	87.05 ± 0.08
60	19.47 ± 0.58	59.37 ± 1.77

**Table 3.** The effect of the ratio of added PEG to Hmim on drug loading.

PEG:Hmim	Drug loading (%)	Encapsulation efficiency (%)
3%	12.46 ± 0.01	82.87 ± 0.08
6%	20.94 ± 0.01	92.12 ± 0.03
12%	15.89 ± 0.05	85.78 ± 0.26

### 3.2. Sample characterization

**Figure 1.**
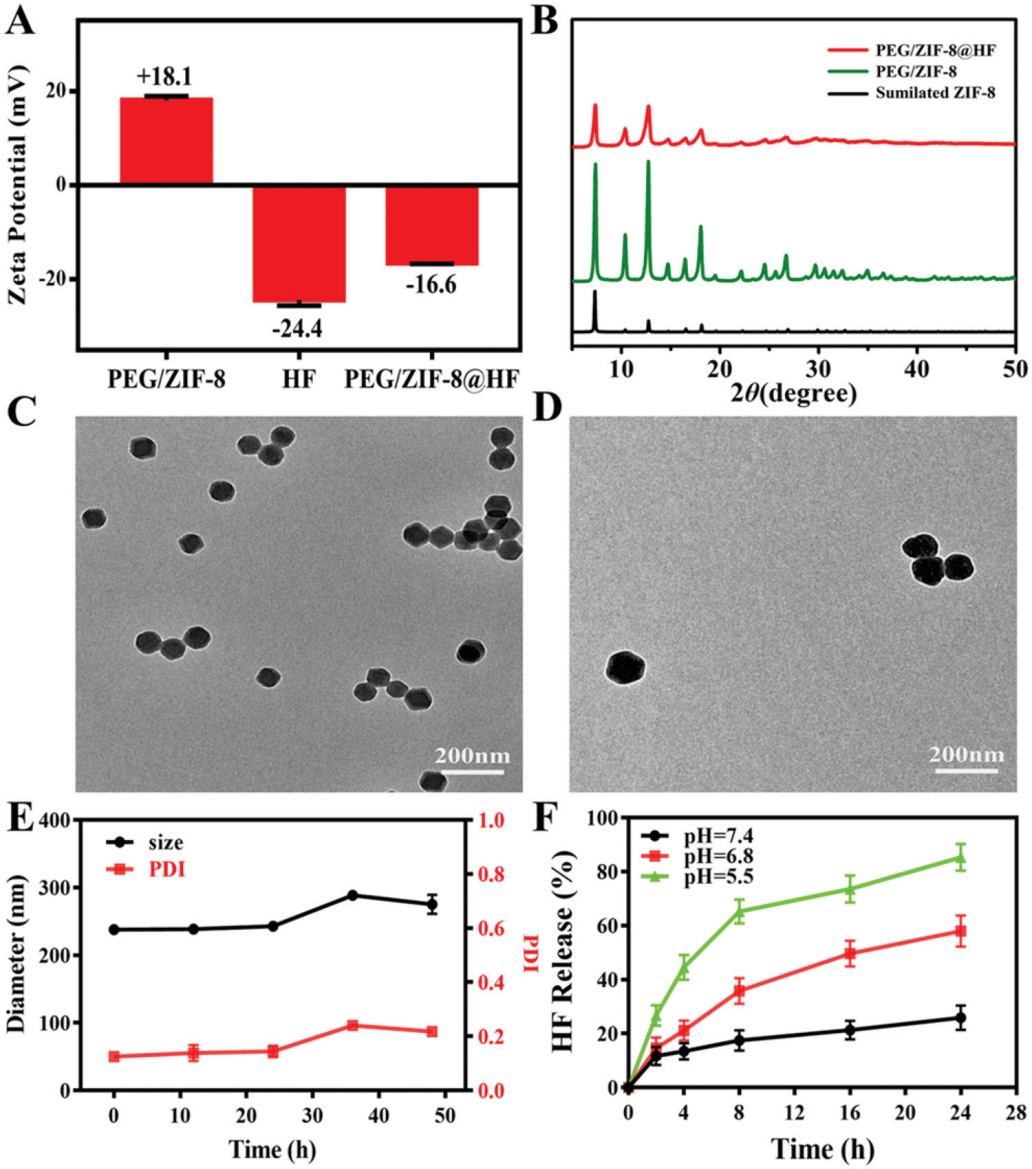
Zeta potentials of PEG/ZIF-8, HF, and PEG/ZIF-8@HF (A); XRD patterns of PEG/ZIF-8, PEG/ZIF-8@HF, and simulated ZIF-8 (B); TEM images of PEG/ZIF-8 (C) and PEG/ZIF-8@HF (D), the scale bar is 200 nm; the change of hydrodynamic diameter and PDI of PEG/ZIF-8@HF in PBS solution (E); HF release curve of PEG/ZIF-8@HF in PBS solutions of different pH values (F).

Table 4 shows the average particle size of various samples. Average particle sizes of ZIF-8, ZIF-8@HF, PEG/ZIF-8, and PEG/ZIF-8@HF are 186.25 nm, 507.15 nm, 170.60 nm, and 228.60 nm, respectively. It indicates that PEG modification can reduce the particle size and drug loading can increase the particle size. PDI values of ZIF-8, ZIF-8@HF, PEG/ZIF-8, and PEG/ZIF-8@HF are 0.11, 0.19, 0.18, and 0.18, respectively. It suggests that PEG/ZIF-8@HF has good dispersion. Zeta potentials of PEG/ZIF-8, HF, and PEG/ZIF-8@HF were detected, and the results are shown in Figure 1(A). Zeta potentials of PEG/ZIF-8, HF, and PEG/ZIF-8@HF are +18.1 mV, −24.4 mV, and −16.6 mV, respectively. The zeta potential of PEG/ZIF-8@HF is located between PEG/ZIF-8 and HF, which indirectly proves that HF is successfully loaded on PEG/ZIF-8. Figure 1(B) shows the XRD patterns of PEG/ZIF-8 and PEG/ZIF-8@HF. Typical XRD patterns of ZIF-8 are observed from PEG/ZIF-8 and PEG/ZIF-8@HF, which indicates that PEG and HF on the samples have no apparent influence on the crystal structure of ZIF-8. Figure 1(C,D) shows the TEM images of PEG/ZIF-8 and PEG/ZIF-8@HF, respectively. A typical morphology of ZIF-8 is observed. The particle sizes and PDI of PEG/ZIF-8@HF after being soaked in PBS for 48 h were tested, and the results are showed in Figure 1(E). The particle size and PDI of PEG/ZIF-8@HF after immersing in PBS for 48 h slightly fluctuate, but there is no significant change. It indicates that PEG/ZIF-8@HF has relatively good stability. Figure 1(F) shows the pH-responsive drug release of HF from PEG/ZIF-8@HF. The average release rates of HF are 25.85%, 57.96%, and 85.31% which correspond to the pH of 7.4, 6.8, and 5.5. This indicates that PEG/ZIF-8@HF shows a higher release rate at a lower pH value, achieving the drug release in response to the acidic microenvironment.

**Table 4.** Particle size distribution spectrum and PDI of ZIF-8, ZIF-8@HF, PEG/ZIF-8, and PEG/ZIF-8@HF.

Group	Particle size (nm)	PDI
ZIF-8	186.25 ± 0.15	0.11 ± 0.01
ZIF-8@HF	507.15 ± 8.35	0.19 ± 0.01
PEG/ZIF-8	170.60 ± 0.70	0.18 ± 0.01
PEG/ZIF-8@HF	228.60 ± 1.40	0.18 ± 0.01

### 3.3. Cell compatibility evaluation

**Figure 2.**
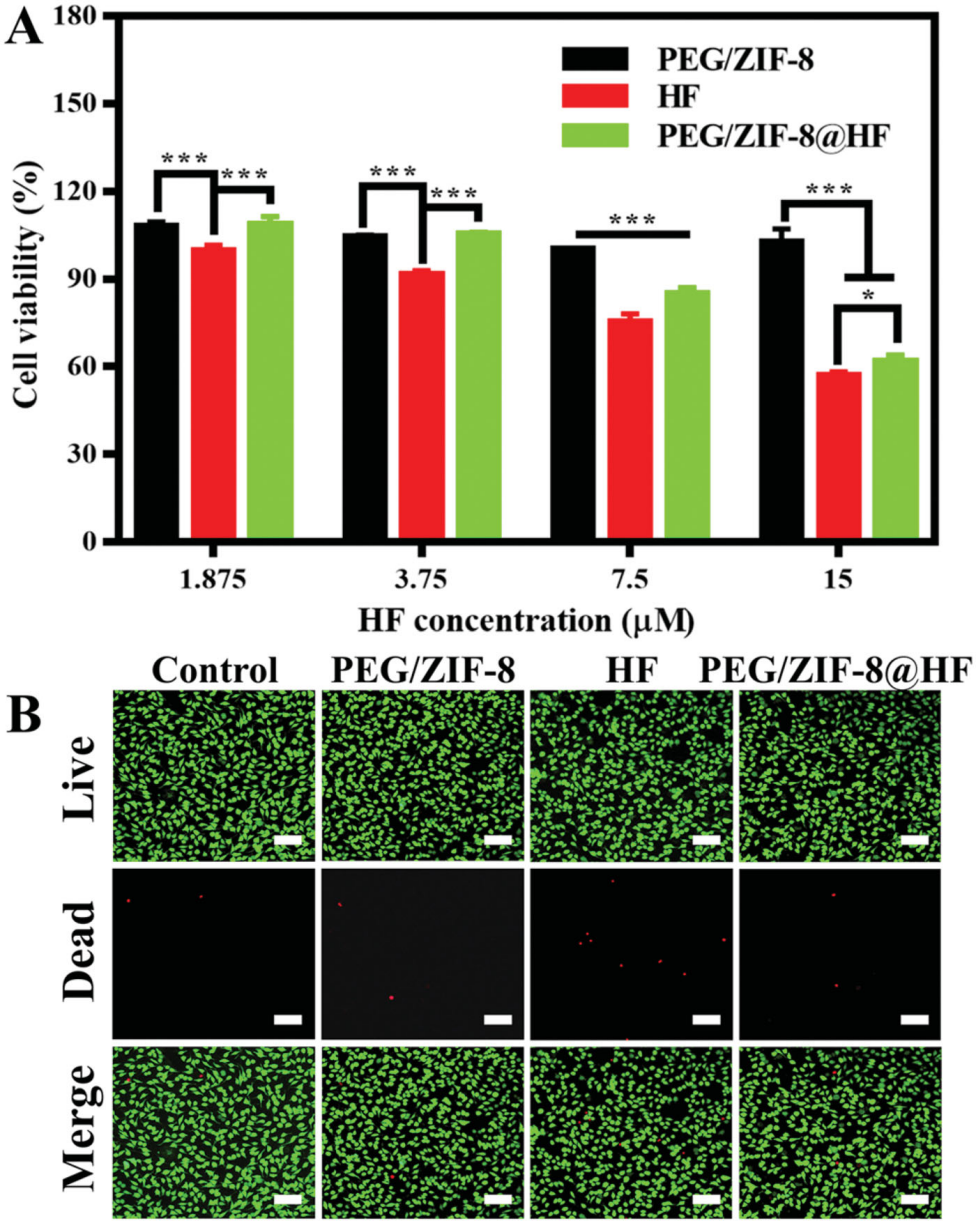
(A) Cell proliferation of L929 cells cultured with PEG/ZIF-8, HF, and PEG/ZIF-8@HF for 24 h, *p<.05, ***p<.001; (B) live/dead cell staining of L929 cells cultured with PEG/ZIF-8, HF, and PEG/ZIF-8@HF for 24 h, the scale bar is 100 µm.

L929 cells are utilized to assess the biocompatibility of PEG/ZIF-8, HF, and PEG/ZIF-8@HF. The alamarBlue test is utilized to assess the cell proliferation rate of PEG/ZIF-8, HF, and PEG/ZIF-8@HF and the results are shown in Figure 2(A). PEG/ZIF-8 with different concentrations all presented good cell viability which were all above 99%, which indicates that the drug-loaded material of PEG/ZIF-8 has good biocompatibility. However, the cytotoxicity of free HF to L929 cells was dose-dependent. The cell viability of L929 cells reduced to 56.80% at the HF concentration of 15 μM. When the concentration of HF was 7.5 μM, the cell viability of L929 cells from PEG/ZIF-8, HF, and PEG/ZIF-8@HF was 100.10%, 75.18%, and 84.62%, respectively. The cell compatibility of PEG/ZIF-8@HF is significantly better than that from free HF. Based on the above results, the concentration of HF with 7.5 μM was utilized in the following single-concentration experiments. Figure 2(B) shows the live/dead staining fluorescent images of L929 cells cultured with PEG/ZIF-8, HF, and PEG/ZIF-8@HF at the HF concentration of 7.5 μM for 24 h. Green fluorescence suggests live cell, and red fluorescence represents dead cell. A large amount of green fluorescence can be observed and the number of red fluorescence is negligible from PEG/ZIF-8, HF, and PEG/ZIF-8@HF. It indicates that PEG/ZIF-8, HF, and PEG/ZIF-8@HF have no obvious cytotoxicity.

**Figure 3.**
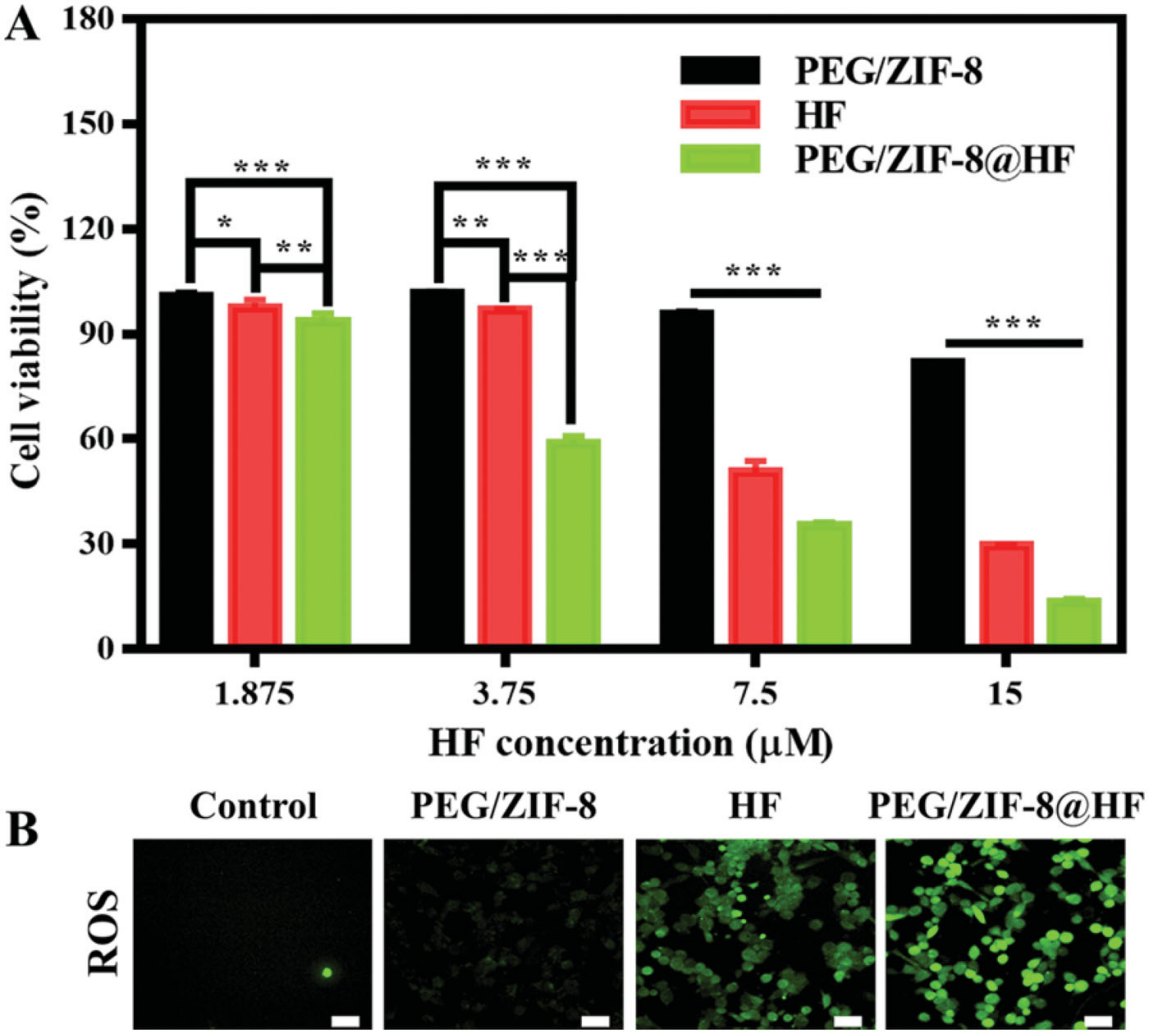
(A) Cell proliferation of B16F10 cells cultured with PEG/ZIF-8, HF, and PEG/ZIF-8@HF for 24 h, *p<.05, **p<.01, and ***p<.001; (B) fluorescence images indicating the ROS levels of B16F10 cells cultured with PEG/ZIF-8, HF, and PEG/ZIF-8@HF for 6 h and detected by DCFH-DA fluorescent probe, the scale bar is 50 µm.

B16F10 cells are used to evaluate the inhibitory effect of PEG/ZIF-8, HF, and PEG/ZIF-8@HF against melanoma *in vitro*. The alamarBlue test is used to evaluate the cytotoxicity of B16F10 cells cultured with PEG/ZIF-8, HF, and PEG/ZIF-8@HF, and the results are presented in Figure 3(A). PEG/ZIF-8 with different concentrations present good cell viability which were all above 80%, indicating that the single material has a weak inhibitory effect on tumor cell growth. However, with the increase of HF concentration, cell viability from HF group and PEG/ZIF-8@HF group reduce, and the cell viability of the PEG/ZIF-8@HF group at each concentration was significantly lower than that of the HF group. It indicates that PEG/ZIF-8@HF shows better inhibitory effect of B16F10 cells than that of HF.

### 3.4. ROS level assessment

Studies have shown that the increase of ROS level in cells leads to cell oxidative stress, which in turn activates the key signal factors of the mitochondrial-dependent apoptosis pathway, thereby promoting the occurrence of apoptosis (Simon et al., 2000; Zhang et al., 2010). HF can significantly up-regulate intracellular ROS levels through ROS-mediated intrinsic mitochondrial pathway to achieve anti-tumor effects (Ou et al., 2017). The ROS levels of PEG/ZIF-8, HF, and PEG/ZIF-8@HF were detected using DCFH-DA probe and the results are presented in Figure 3(B). Green fluorescence indicates the ROS level in the cell. Green fluorescence from PEG/ZIF-8 is negligible, which suggests that the intracellular ROS level is very low. The fluorescence intensity of cells cultured with HF was enhanced significantly but weaker than those treated with PEG/ZIF-8@HF. Yin et al. detected the HF content in the plasma of male rats after a single intravenous administration of 1.0 mg/kg HF, and the experiment showed that after administration, HF exhibited biexponential clearance kinetics. The half-life of drug elimination (*t*_1/2_) at the terminal phase was 6.10 ± 1.86 h, which indicated that the stability of free HF was extremely poor (Yin et al., 2017). Therefore, the fluorescence intensity of PEG/ZIF-8@HF group is significantly stronger than that of the free HF group, which may be due to the fact that PEG/ZIF-8@HF can completely encapsulate the drug and release it slowly, achieving a better anti-tumor effect than the unstable free HF.

### 3.5. Migration and invasion of B16F10 cells

**Figure 4.**
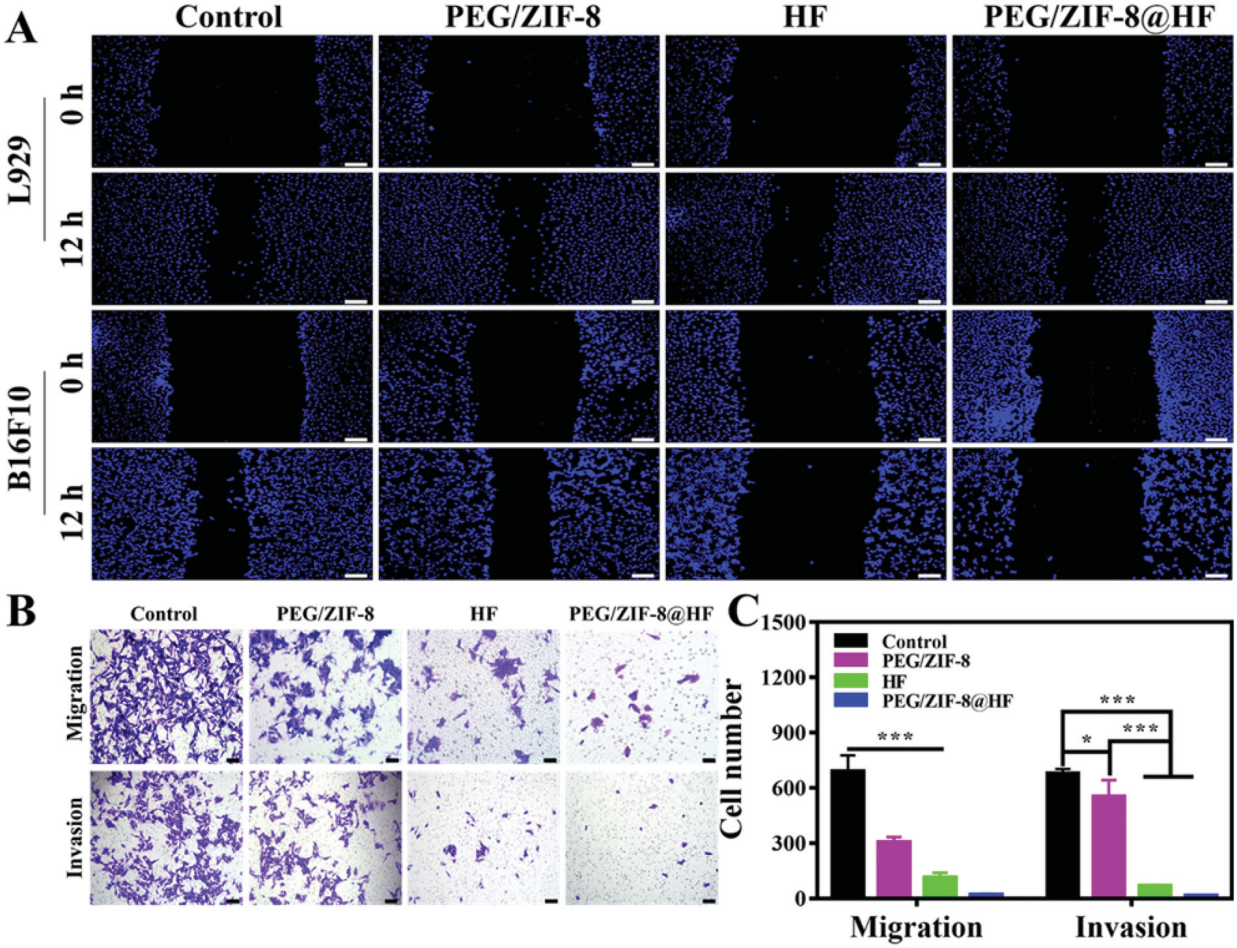
Scratch healing ability of L929 cells and B16F10 cells cultured on PEG/ZIF-8, HF, and PEG/ZIF-8@HF, the scale bar is 100 µm (A); migration and invasion ability of B16F10 cells cultured on PEG/ZIF-8, HF, and PEG/ZIF-8@HF, the scale bar is 50 µm (B), and the corresponding quantitative analysis, *p<.05, ***p<.001 (C).

The migration and invasion of tumor cells are one of the main causes of cancer-related death (Chen, 2013). Therefore, the scratch test, and the migration and invasion tests were utilized to assess the effects of PEG/ZIF-8, HF, and PEG/ZIF-8@HF on the migration and invasion of L929 cells and B16F10 cells. Figure 4(A) shows the results of the scratch test. Compared with the control group, PEG/ZIF-8 and PEG/ZIF-8@HF had almost no impact on the migration of L929 cells, and HF showed a weak inhibitory effect on the migration of L929 cells. For B16F10 cells, PEG/ZIF-8 had no apparent influence on its migration ability compared with control group. However, HF and PEG/ZIF-8@HF exhibited obvious inhibitory effects. At the same time, as shown in Figure 4(B,C), under a fixed field of view, the average number of cells migrated in the control, PEG/ZIF-8, HF, and PEG/ZIF-8@HF groups were 690, 305, 112, and 21, respectively. While the average number of cells invaded are 677, 553, 67, and 14 which correspond to the control, PEG/ZIF-8, HF, and PEG/ZIF-8@HF, respectively. Compared with control group, PEG/ZIF-8@HF showed the greatest inhibitory effect on the migration and invasion of B16F10 cells, followed by HF and PEG/ZIF-8. It suggests that HF encapsulated in PEG/ZIF-8 (PEG/ZIF-8@HF group) at same concentration shows better inhibitory effect that HF alone.

### 3.6. Anti-tumor efficacy *in vivo*

**Figure 5.**
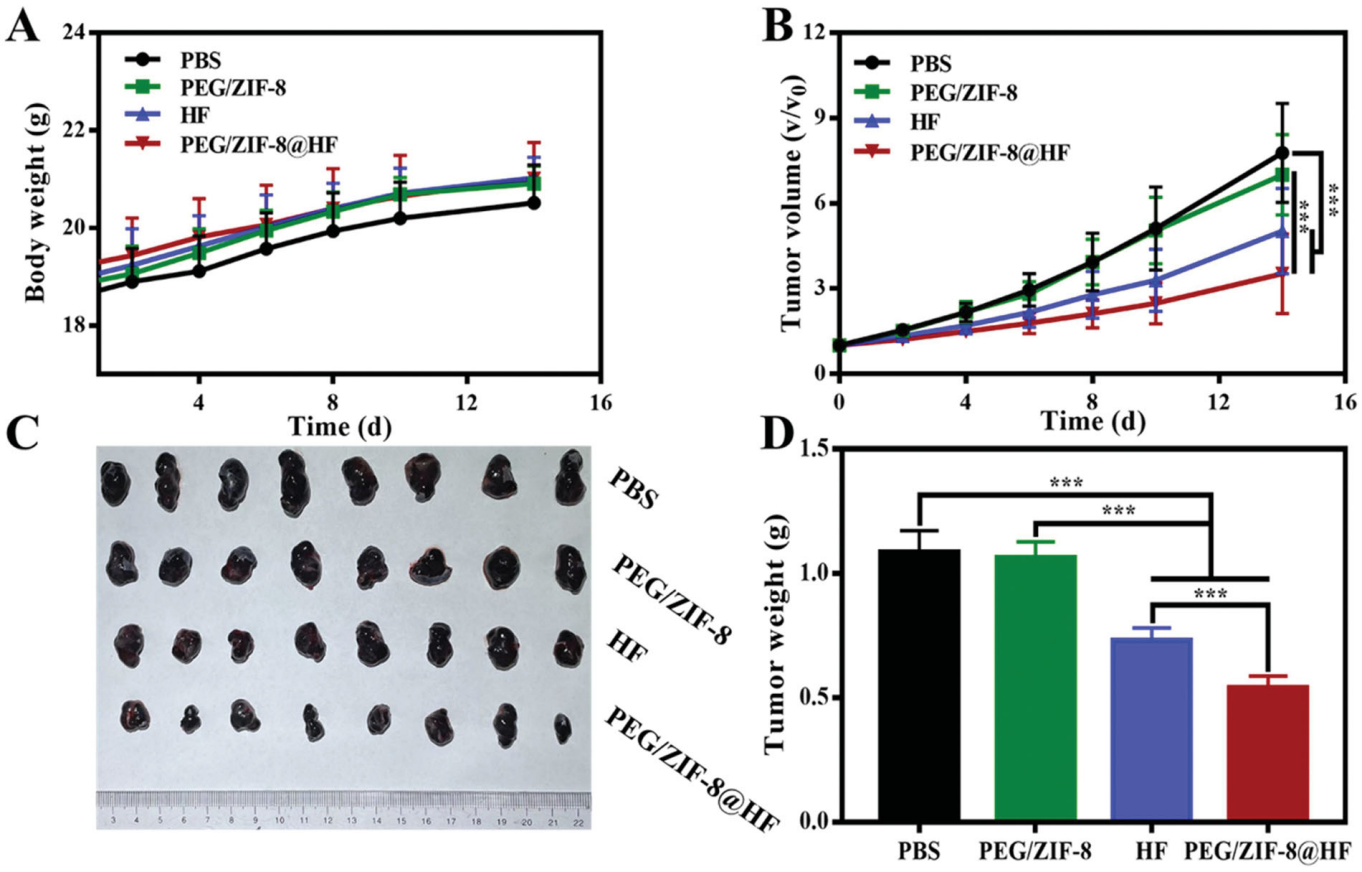
(A) Body weight change curve of tumor-bearing nude mice; (B) tumor volume changes of Control, PEG/ZIF-8, HF, and PEG/ZIF-8@HF groups, ***p<.001; (C) photographs of tumors taken from control, PEG/ZIF-8, HF, and PEG/ZIF-8@HF groups and (D) the corresponding quantitative analysis of tumor weight, ***p<.001.

The anti-tumor effect of PEG/ZIF-8@HF was studied in B16F10 tumor-bearing nude mice. As indicated in Figure 5(A), the weight of nude mice from various groups had no significant difference during different timeframes. Figure 5(B) shows the 14 days tumor growth curves of nude mice in different groups. It could be found that the tumor volume of PEG/ZIF-8@HF was significantly smaller than that of the other groups on day 14. Photographs of tumors from various groups on the 14th day are presented in Figure 5(C) and it further confirms that PEG/ZIF-8@HF had the smallest tumor volume. Corresponding tumor weight was measured and the results are shown in Figure 5(D). The average tumor weights of PBS, PEG/ZIF-8, HF, and PEG/ZIF-8@HF were 1.09 g, 1.06 g, 0.73 g, and 0.54 g, respectively. It suggests that PEG/ZIF-8@HF group has the best anti-tumor effects against melanoma.

**Figure 6.**
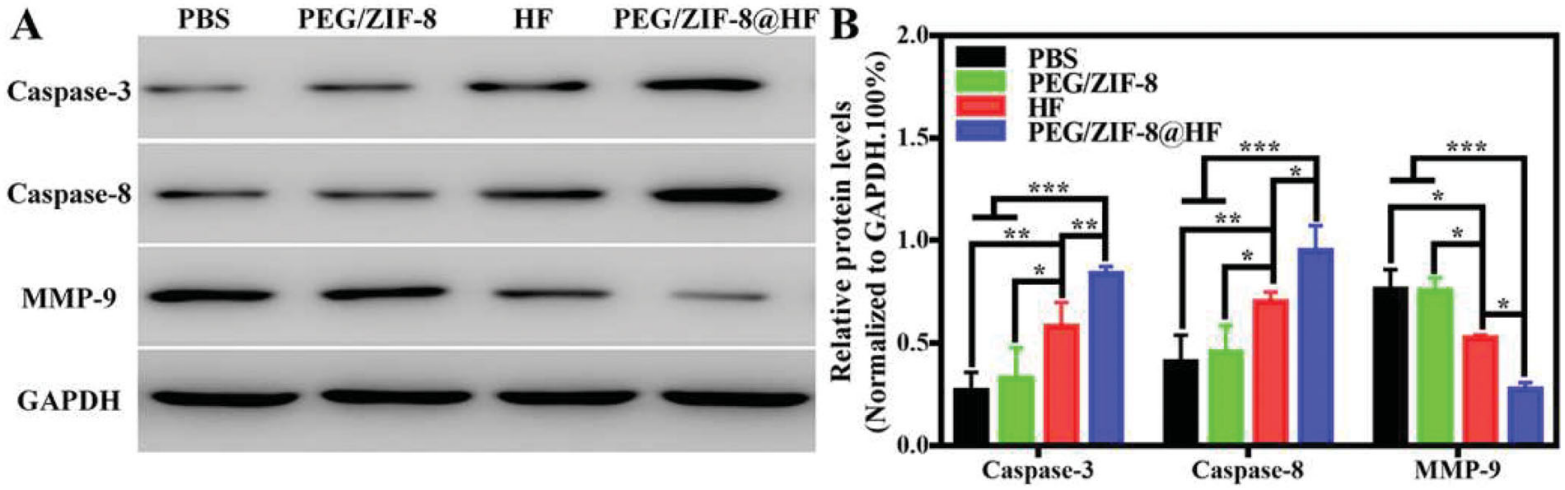
Western blot detection of the expressions of caspase-3, caspase-8, and MMP-9 proteins in the tumor tissues from PBS, PEG/ZIF-8, HF, and PEG/ZIF-8@HF groups (A), and the corresponding quantitative analysis, *p<.05, **p<.01, and ***p<.001 (B).

Studies have shown that caspase-8 and caspase-3 are important biological enzymes in the exogenous and endogenous apoptotic pathways, respectively, which can induce apoptosis (Abdel Shakor et al., 2015). It has been reported that HF can rely on multiple van der Waals contacts and hydrogen bonds to form a pharmacophore with the S1 active site of MMP-9, which reduces its activity to inhibit tumor cell migration and invasion (Kalva et al., 2014). Western blot was utilized to analyze the expressions of caspase-3, caspase-8, and MMP-9 in tumor tissues, and the results are presented in Figure 6(A). Compared to PBS group, the expressions of these three proteins in PEG/ZIF-8 had no significant difference. However, caspase-3 and caspase-8 bands from PEG/ZIF-8@HF were thicker than those from HF, and the MMP-9 band from PEG/ZIF-8@HF is thinner than that from HF. The relative quantitative protein levels of caspase-3, caspase-8, and MMP-9 are shown in Figure 6(B). Compared with HF, PEG/ZIF-8@HF significantly enhanced the expressions of caspase-3 and caspase-8, and inhibited the activity of MMP-9. This indicates that HF encapsulated in PEG/ZIF-8@HF exhibits better anti-tumor effect which is closely related to the up-regulation expressions of caspase-3, caspase-8, and down-regulation expression of MMP-9.

**Figure 7.**
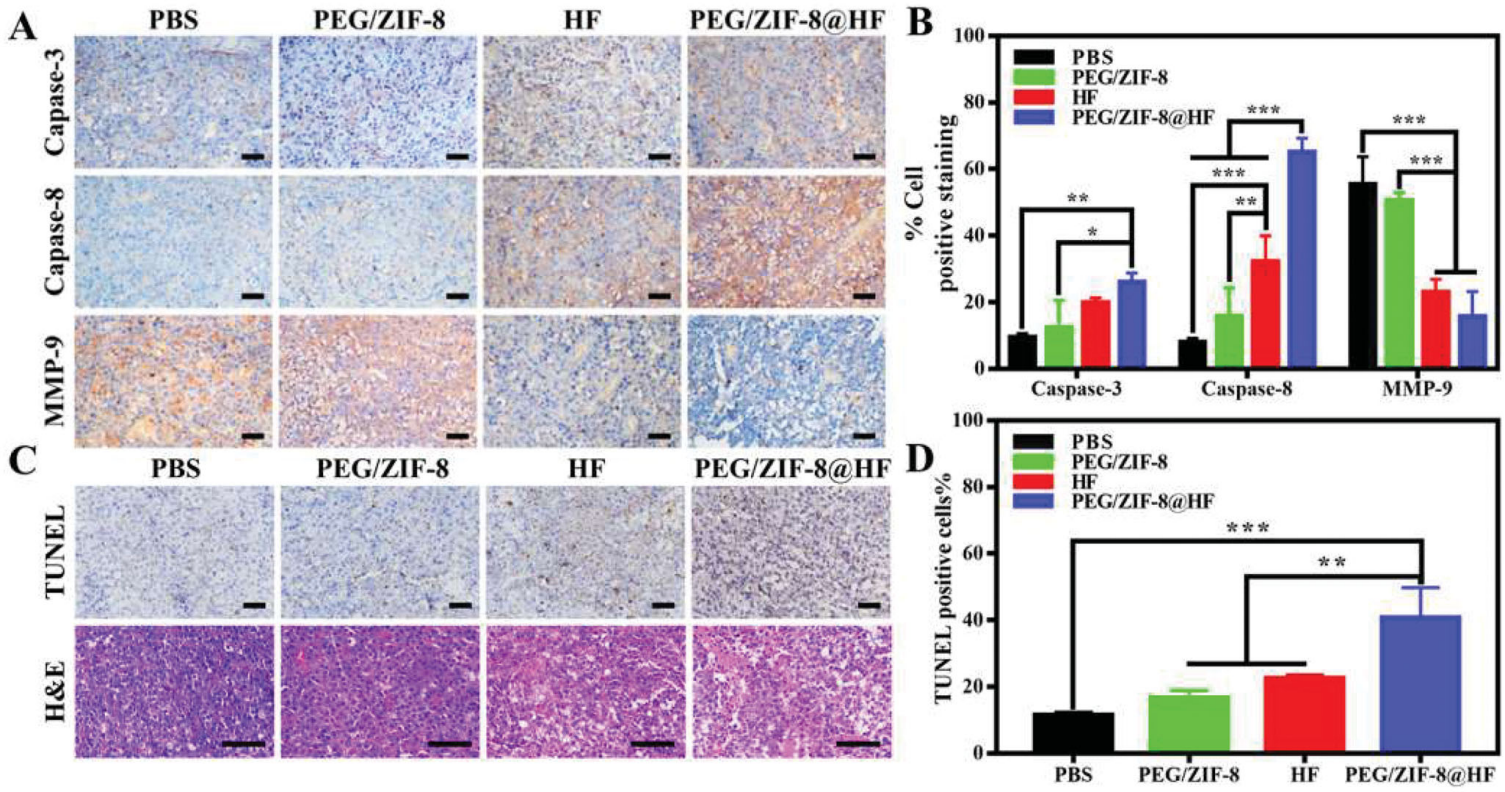
Immunohistochemical analysis of caspase-3, caspase-8, and MMP-9 in tumor slices from PBS, PEG/ZIF-8, HF, and PEG/ZIF-8@HF groups, the scale bar is 50 µm (A), and the corresponding quantitative analysis of positive staining cells, *p<.05, **p<.01, and ***p<.001 (B); TUNEL (the scale bar is 50 µm) and HE (the scale bar is 100 µm) staining images of tumor sections from PBS, PEG/ZIF-8, HF, and PEG/ZIF-8@HF groups (C), and the corresponding quantitative analysis of positive staining cells based on the TUNEL staining images, **p<.01 and ***p<.001 (D).

**Figure 8.**
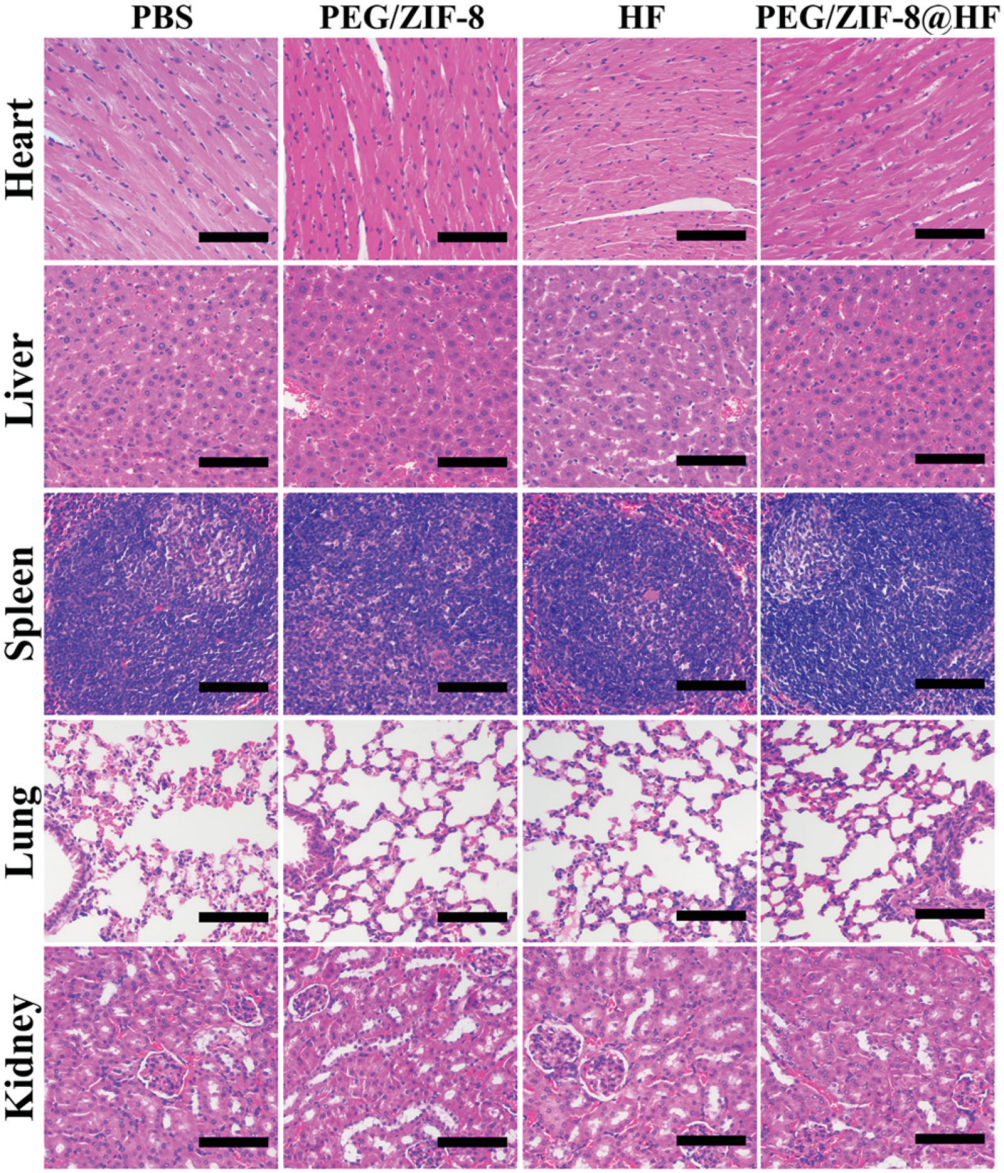
H&E staining images of the heart, liver, spleen, lung, and kidney from PBS, PEG/ZIF-8, HF, and PEG/ZIF-8@HF groups, the scale bar is 100 µm.

Immunohistochemical staining images from various groups are presented in Figure 7(A) and the corresponding quantitative result is presented in Figure 7(B). Compared with PEG/ZIF-8 and HF, the tumor sections from PEG/ZIF-8@HF showed a significant increase in the expressions of caspase-3 and caspase-8 positive cells and a decrease in the expression of MMP-9 positive cells. TUNEL and HE staining images of tumor sections from PBS, PEG/ZIF-8, HF, and PEG/ZIF-8@HF are presented in Figure 7(C), and the corresponding quantitative results of positive staining cells based on the TUNEL staining images are shown in Figure 7(D). TUNEL positive cells from PEG/ZIF-8@HF significantly increased. The average apoptosis rate from PEG/ZIF@HF was 40.83%, which was much higher than those of 11.53%, 16.73%, and 22.43% from PBS, PEG/ZIF-8, and HF, respectively. The results of HE staining indicated that, compared with the closely arranged tumor tissues in the PBS group, the tumor tissues in the PEG/ZIF-8@HF group had a large number of nuclear pyknosis, and the apoptotic bodies and cell structures disappeared completely. The degrees of tumor cell shrinkage distortion and dissolves from PEG/ZIF-8@HF were significantly higher than those from PEG/ZIF-8 and HF groups. It indicates that PEG/ZIF-8@HF enhances the anti-melanoma effect of free HF. Figure 8 shows the HE staining result of heart, liver, spleen, lung, and kidney. It showed no apparent organ abnormalities or lesions from each experimental group compared with the PBS group.

HF is an important active ingredient isolated from *Selaginella tamariscina* Spring (Shim et al., 2018). In the past few decades, there have been a great number of studies on the anti-tumor pharmacological activity of HF. Yang et al. reported that HF could effectively disrupt cell migration and invasion by enhancing the expression of ROS, lowering the mitochondrial membrane potential, and down-regulating the expression of MMP-2 and MMP-9 to inhibit the proliferation of human melanoma A375 and CHL-1 cells (Yang et al., 2018). It indicates that HF is expected to become a new chemotherapeutic drug for the treatment of melanoma. However, HF is difficult to dissolve in water, resulting in low bioavailability and high oral dose, which greatly limits its clinical application (Chen et al., 2019). In this work, HF was encapsulated into ZIF-8 and grafted with polyethylene glycol to fabricate drug-delivery system of PEG/ZIF-8@HF. PEG/ZIF-8@HF shows enhanced water solubility, high drug loading (20.94%) and encapsulation efficiency (92.12%) which are higher than those of the HF hybrid nanomicelle (Chen et al., 2020). Moreover, PEG/ZIF-8@HF can realize drug release in response to an acidic microenvironment. Compared with free HF, PEG/ZIF-8@HF shows better biocompatibility against L929 cells while more effectively inhibitory efficiency against B16F10 cells. It may be ascribed to the higher release rate of PEG/ZIF-8@HF in acidic tumor environment. Besides, PEG/ZIF-8@HF can produce a higher ROS level and show up-regulating the pro-apoptotic proteins caspase-3 and caspase-8, and down-regulating the migration-promoting invasion protein MMP-9 which lead to good anti-tumor effect against melanoma *in vitro* and *in vivo.*

## 4. Conclusions

In this work, HF was encapsulated in ZIF-8 grafted with PEG through a one-step synthesis method and the composite material is denoted as PEG/ZIF-8@HF. PEG/ZIF-8@HF has a high encapsulation efficiency and can achieve selective drug release in an acidic microenvironment. The results of *in vitro* anti-melanoma experiments indicate that PEG/ZIF-8@HF shows up-regulation of ROS levels and can inhibit the migration and invasion of B16F10 cells. Besides, animal experiments *in vivo* via oral administration of PEG/ZIF-8@HF further confirms that PEG/ZIF-8@HF shows anti-tumor effect by up-regulating the pro-apoptotic proteins caspase-3 and caspase-8, and down-regulating the migration-promoting invasion protein MMP-9. This study developed a safe and effective oral administration of HF based on the high-efficiency delivery ZIF-8 system, which provides an effective treatment strategy for melanoma.
